# Differential Contributions of mSWI/SNF Chromatin Remodeler Sub-Families to Myoblast Differentiation

**DOI:** 10.3390/ijms241411256

**Published:** 2023-07-09

**Authors:** Teresita Padilla-Benavides, Monserrat Olea-Flores, Tapan Sharma, Sabriya A. Syed, Hanna Witwicka, Miriam D. Zuñiga-Eulogio, Kexin Zhang, Napoleon Navarro-Tito, Anthony N. Imbalzano

**Affiliations:** 1 Department of Molecular Biology and Biochemistry, Wesleyan University, Middletown, CT 06459, USA; monserrat.oleaflores@umassmed.edu (M.O.-F.); miriamzuniga@uagro.mx (M.D.Z.-E.); kzhang@wesleyan.edu (K.Z.); 2 Department of Biochemistry and Molecular Biotechnology, University of Massachusetts Chan Medical School, Worcester, MA 01605, USA; tapan.sharma@umassmed.edu (T.S.); sabriya.syed@jax.org (S.A.S.); hanna.witwicka@crl.com (H.W.); 3 Facultad de Ciencias Químico Biológicas, Universidad Autónoma de Guerrero, Chilpancingo de los Bravo 39086, GRO, Mexico; nnavarro@uagro.mx

**Keywords:** SWI/SNF, myogenesis, Baf250A, Brd9, myogenin, gene regulation, chromatin remodeling

## Abstract

Mammalian SWI/SNF (mSWI/SNF) complexes are ATP-dependent chromatin remodeling enzymes that are critical for normal cellular functions. mSWI/SNF enzymes are classified into three sub-families based on the presence of specific subunit proteins. The sub-families are Brm- or Brg1-associated factor (BAF), ncBAF (non-canonical BAF), and polybromo-associated BAF (PBAF). The biological roles for the different enzyme sub-families are poorly described. We knocked down the expression of genes encoding unique subunit proteins for each sub-family, *Baf250A*, *Brd9*, and *Baf180*, which mark the BAF, ncBAF, and PBAF sub-families, respectively, and examined the requirement for each in myoblast differentiation. We found that Baf250A and the BAF complex were required to drive lineage-specific gene expression. KD of *Brd9* delayed differentiation. However, while the Baf250A-dependent gene expression profile included myogenic genes, the Brd9-dependent gene expression profile did not, suggesting Brd9 and the ncBAF complex indirectly contributed to differentiation. Baf180 was dispensable for myoblast differentiation. The results distinguish between the roles of the mSWI/SNF enzyme sub-families during myoblast differentiation.

## 1. Introduction

SWI/SNF chromatin remodelers are ATP-dependent enzymes that alter nucleosome structure to facilitate or prevent access of regulatory factors to the genome and are conserved throughout the eukaryotic kingdom [1,2,3,4]. Early work in yeast determined that genes encoding proteins involved in mating type switching (SWI) and in sucrose fermentation (SNF) formed a multi-protein complex containing a DNA-dependent ATPase of the SNF2 family [5,6]. Subsequent work in both yeast and human cells determined that such complexes altered the structure of nucleosomes in an ATP-dependent manner and promoted binding of transcription factors to the nucleosome [7,8,9,10]. Formal demonstration of enzymatic activity followed [11]. Studies of SWI/SNF complexes in mammalian cells revealed that significant diversity exists in the assembly of the complexes [12,13,14,15,16]. There are two mutually exclusive ATPases, called Brahma (Brm) and Brahma-related gene 1 (Brg1 [17,18]). The original purifications of mammalian SWI/SNF complexes identified two chromatographically separable fractions, originally termed “A” and “B”, that contained ATP-dependent nucleosome remodeling activity that tracked with the presence of the ATPase protein [8,9,10]. These complexes were subsequently renamed Brg1- or Brm-associated factor (BAF) and polybromo-associated BAF (PBAF), respectively, based not on the ATPase responsible for enzymatic activity but on the presence of subunits that were unique to each complex [19].

Additional work has shown that specific mSWI/SNF subunit proteins have different splice forms, can be encoded by multiple genes that are differentially expressed among tissue types or under different conditions, and consequently can be assembled into many distinct complexes that differ in subunit composition and function. Based on the number of identified subunits and their variants, in theory there can be thousands of combinations that can constitute a mammalian SWI/SNF (mSWI/SNF) complex [12,13,16]. Nevertheless, the diverse compositions of complexes could still be classified as members of the BAF or PBAF families. More recently, a third sub-family of complex has been recognized, called non-canonical BAF (ncBAF). ncBAF lacks some of the subunits that are common to BAF, and PBAF and has other subunits not found in those two sub-families [20,21,22,23,24]. 

The catalytic activity of Brg1/Brm and homologs form other species share a common mechanism by which ATP-hydrolysis powers alteration of the path and position of the DNA around a nucleosome particle [1,2,3,4]. Consequently, the diversity of mSWI/SNF complexes likely originated from the need to target the catalytic activity to different places in the genome, as well as provide specialized functions. 

Research related to mSWI/SNF contributions towards skeletal muscle differentiation has been focused on the requirement and roles for the catalytic subunit. For instance, ectopic expression of catalytically inactive Brg1 or Brm subunits inhibited differentiation, prevented chromatin remodeling of several myogenic genes, and consequently decreased their expression [25,26,27,28,29,30,31]. Brg1 and Brm have differential roles in regulating gene expression during myogenesis. Brg1 is required for the transcription of myogenic genes at early stages of differentiation, while Brm is required for *Ccnd1* repression and cell cycle arrest that precedes the activation of late muscle genes [31]. Core subunits shared by BAF, PBAF, and ncBAF complexes have been also implicated in the development of heart and skeletal muscle development during mouse embryogenesis [32,33,34,35]. Among these, the Baf60c subunit has a critical role in targeting mSWI/SNF enzymes to myogenic promoters [27,30]. Baf53a, another core component of the three sub-families, and Snf5, a subunit shared only by BAF and PBAF complexes, are also required for the transcriptional activation of myogenic genes [36].

mSWI/SNF complexes are also essential for myoblast proliferation. Brg1 and Snf5 subunits contribute to myoblast cell cycle progression, and knockout (KO) of *Brg1* in primary myoblasts resulted in cell death [36,37]. Brg1 is essential for *Pax7* expression, a master transcription factor that supports myoblast proliferation and survival [37]. The regulation of *Pax7* expression is partially regulated by casein kinase 2-mediated phosphorylation of Brg1 [38]. Moreover, short hairpin RNA (shRNA) knockdown (KD) of distinguishing subunits of the BAF, ncBAF, and PBAF complexes in C2C12 myoblasts showed the differential contributions of each sub-family to myoblast proliferation. KD of specific subunits of the BAF or the ncBAF complexes reduced myoblast proliferation rate, while KD of PBAF-specific subunits did not affect proliferation [39]. RNA-seq analyses from proliferating myoblasts knocked down for *Baf250A* (BAF complex) exhibited a reduction in *Pax7* expression due to a decreased binding of Baf250A and impaired chromatin remodeling at the promoter of this proliferation marker. The proliferation defect was reversed by reconstituting Pax7 expression using a doxycycline-inducible lentiviral vector. Thus, the work demonstrated that the BAF sub-family is required for myoblast proliferation via regulation of Pax7 expression [39]. 

In the current work, we aimed to understand the specific contributions of BAF, ncBAF, and PBAF enzymes to myoblast differentiation, using a similar strategy of shRNA-mediated KD of the expression of the individual mSWI/SNF enzyme subunits that are specific to the three sub-families (*Baf250A*, *Brd9*, and *Baf180*, respectively [39]). Our data show that the BAF complex is required for myoblast differentiation as it regulates the expression of *myogenin* and other myogenic genes required to initiate and continue the myogenic program. Mechanistic analyses determined that Baf250A is bound to the regulatory sequences controlling the expression of these genes. RNA-seq and subsequent GO analyses of differentially expressed genes in *Brd9* KD differentiating myoblasts did not identify terms associated with skeletal muscle, suggesting that the ncBAF complexes contributed to myogenesis indirectly. The PBAF sub-family was verified to be dispensable for myoblast differentiation as was previously reported [40,41,42]. Our work provides functional and mechanistic details of the differential regulatory roles of the different sub-families of mSWI/SNF enzyme complex during skeletal muscle differentiation and supports a role for the BAF complex as an essential contributor to this process.

## 2. Results

### 2.1. The BAF Sub-Family of mSWI/SNF Complexes Is Required for Myoblast Differentiation In Vitro

**Figure 1:**
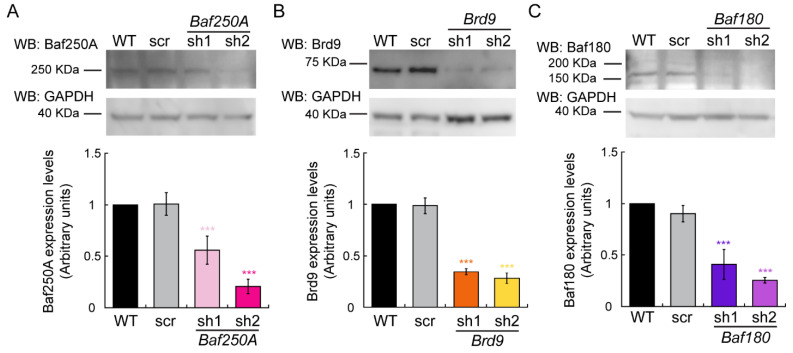
Baf250A, Brd9, and Baf180 expression in wild-type (WT), shRNA scrambled (scr) control, and the indicated shRNA-mediated knockdowns in differentiating C2C12 myoblasts. Representative Western (**top**) and quantification (**bottom**) of Baf250A (**A**), Brd9 (**B**), and Baf180 (**C**) levels in differentiating cells after 48 h of differentiation. Data represents the mean ± SE of three independent biological replicates. GAPDH was used as the loading control. Quantification of each sample was compared to the corresponding wild-type (WT) sample, which was set to 1.0. *** *p* < 0.001.

**Figure 2:**
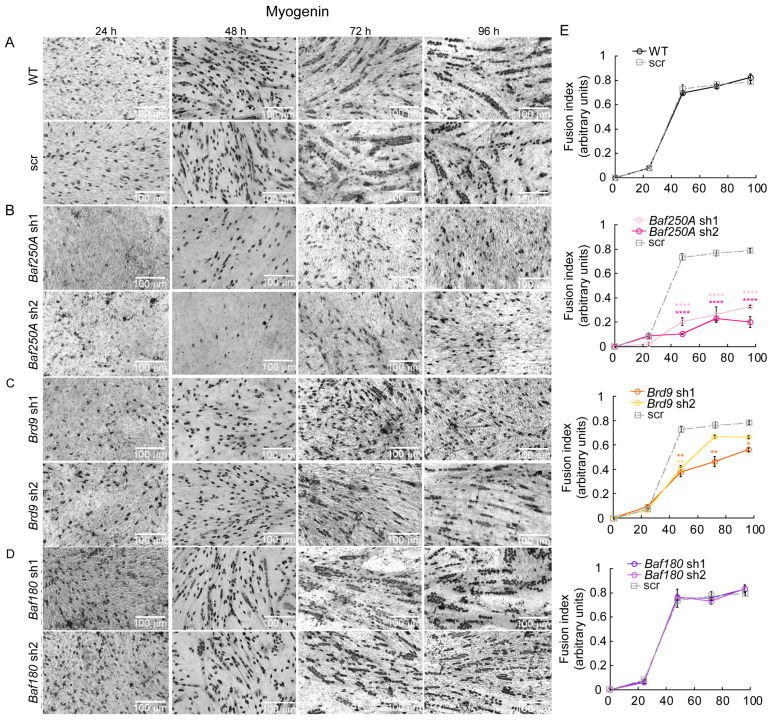
Baf250A knockdown inhibited the differentiation of C2C12 cells. Representative light micrographs of wild-type C2C12 myoblasts (**A**) or myoblasts transduced with scr (**A**), *Baf250A* (**B**), *Brd9* (**C**), or *Baf180* (**D**) shRNAs undergoing differentiation for 24, 48, 72, and 96 h. Cells were immunostained for myogenin. Bars = 100 µm. (**E**) Fusion indices were measured for the indicated samples at 24, 48, 72, and 96 h of differentiation. * *p* < 0.05, ** *p* < 0.01, **** *p* < 0.0001.

Evaluation of proliferating primary murine myoblasts and immortalized C2C12 myoblasts identified Brg1 as the relevant mSWI/SNF ATPase and the BAF sub-family of mSWI/SNF complexes as the major contributors to maintenance of the proliferative state by regulating the expression of *Pax7* [37,39]. We continued our work to determine the roles of the three families of mSWI/SNF complex in the differentiation of myoblasts in vitro. We used a lentiviral system to KD specific subunit proteins that are unique for each of the different sub-families of mSWI/SNF complexes and induced the myoblasts to differentiate. Western blot analyses showed that differentiating C2C12 myoblasts transduced with lentiviral particles containing shRNAs against *Baf250A*, *Brd9*, and *Baf180*, which encode subunits that are unique to the BAF, ncBAF, and PBAF complexes, respectively, showed reduced expression of these proteins (Figure 1). We then assessed the functional effects of these KDs on myoblast differentiation using changes in fusion index as a quantitative measure of the progression of myogenesis. Figure 2 and Figure S1A show representative micrographs of control and KD C2C12 myoblasts undergoing differentiation for 24, 48, 72, and 96 h that were immunostained for myogenin and myosin heavy chain (MHC) expression, respectively. Myogenin and MHC expression in wild-type (untreated) and scrambled sequence shRNA (scr) controls were detected at 24 h after inducing differentiation; myoblast fusion was detected as early as 48 h and peaked at 96 h (Figure 2A and Figure S1A). Myoblasts partially depleted of Baf250A (BAF complex; Figure 2B and Figure S1B) failed to differentiate as shown by a decrease in the expression of myogenin and MHC and a reduction in the fusion index when compared to control cells at similar time points (Figure 2A and Figure S1A). The KD of the *Brd9* subunit unique to the ncBAF complex (Figure 2C and Figure S1C) resulted in a delay in the differentiation progression, as shown by a decrease in expression of differentiation markers and longer times required for these myoblasts to fuse. However, the *Brd9* KD myoblasts were able to differentiate within the 96 h analyzed (Figure 2C and Figure S1C). Partial depletion of Baf180 subunit that is unique to the PBAF complex had no effect on myogenesis, confirming previous results from our group and others that showed that it is dispensable for skeletal muscle differentiation (Figure 2D and Figure S1D [40,41,42]). The data supports a fundamental role for the BAF complex in myoblast differentiation.

### 2.2. KD of *Baf250A* or *Brd9* Elicited Differential Effects in Gene Transcription in Differentiating C2C12 Myoblasts 

**Figure 3:**
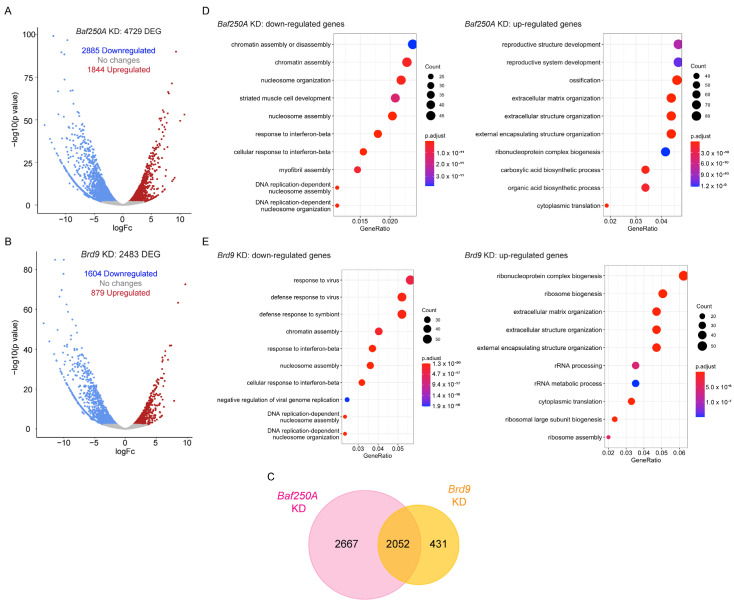
Changes in gene expression dependent on *Baf250A* or *Brd9* knockdown. Volcano plots displaying differentially expressed genes between *scr* control and *Baf250A* (**A**) or *Brd9* (**B**) knockdown C2C12 cells at 48 h of differentiation. The *y*−axis corresponds to the mean log10 expression levels (*p* values). The red and blue dots represent the up- and downregulated transcripts in knockdown cells (false-discovery rate (FDR) of <0.05), respectively. The gray dots represent the expression levels of transcripts that did not reach statistical significance (FDR of >0.05). (**C**) Venn diagram showing the overlapping differentially expressed genes between the differentiating myoblasts knocked down for the *Baf205A* and *Brd9* subunits. GO term analysis of differentially expressed genes in differentiating C2C12 cells knocked down for *Baf250A* (**D**) or *Brd9* (**E**). Cut-off was set at 2.0 of the −log (adjusted *p* value). See Table S2 for the complete list of genes.

We have previously shown that KD of subunits unique to the three different sub-families of the mSWI/SNF complexes had minor effects on gene expression in proliferating myoblasts [39]. In that report, one of the most striking results was the effect of *Baf250A* KD in reducing the expression of *Pax7*, the master regulator of myoblast growth, which partially explained the proliferation deficiency observed in those cells [39]. KD of *Baf250A* also impaired myoblast differentiation, suggesting a primary role of the BAF complex in this process. To better understand the phenotypes observed in myogenesis at a transcriptional level, we performed RNA-seq analyses in differentiating myoblasts transduced with scramble (Scr) shRNA and myoblast expressing shRNAs against *Baf250A* or *Brd9* (Figure 3). In all cases, the sequenced libraries had approximately 45 M total reads. Pearson coefficients were >0.96 for the replicate RNA-seq datasets for each KD and are shown in Table S1. The data were mapped to the mouse genome (mm10), and changes in gene expression were determined. Table S2 shows the differentially expressed genes that showed significant changes in both replicates for each shRNA (log2FoldChange < 1, padj < 0.05). *Baf250A* KD resulted changes in the expression of 4729 genes when compared to control cells; of these, 1844 genes were upregulated, and 2885 were downregulated. (Figure 3A, Table S2). *Brd9* KD affected 2483 genes, of which 879 were upregulated and 1604 downregulated (Figure 3B, Table S2). In addition, we determined there were 2052 genes that were altered in both *Baf250A* and *Brd9* KD cells when compared to control, which represents ~83% of the genes differentially regulated by Brd9. In contrast, only ~43% of the differentially regulated genes in *Baf250A* KD cells were shared with those genes differentially regulated by *Brd9* KD (Figure 3C). These results suggest that there are relatively few genes uniquely regulated by Brd9 in differentiating myoblasts.

Gene ontology (GO) analysis of these differentially expressed genes identified functional categories. GO analyses of *Baf250A* KD differentiating myoblasts determined a deficiency in the expression of genes related to chromatin regulation and nucleosome assembly, which would be expected if a chromatin remodeling enzyme were compromised. Other terms included response to interferon-β and muscle development and structure (Figure 3D). Upregulated genes were related to reproductive system development, ossification, and extracellular matrix organization (Figure 3D). Increases in bone-specific gene expression upon inhibition of muscle-specific gene expression are consistent with the capacity of C2C12 cells to differentiate along osteoblast lineage when exposed to appropriate signaling and may implicate Baf250A as a regulator both lineages [43]. Genes involved in ECM and the extracellular environment are known to be regulated by Brg1 [44,45,46,47]. *Brd9* KD myoblasts showed decreased expression of genes involved in responses to virus, response to interferon-β signaling, and chromatin regulation, similar to the results from the *Baf250A* KD (Figure 3E). Notably, terms related to muscle formation or function were absent, suggesting that only Baf250A, and by extension the BAF complex, regulates muscle-specific genes. The top terms describing genes with enhanced expression upon *Brd9* KD were related to ribonucleoprotein and ribosomal synthesis and extracellular matrix organization (Figure 3E), demonstrating some overlap with the terms upregulated in response to *Baf250A* KD. 

### 2.3. BAF Complex-Mediated Regulation of Myogenic Genes

Dpf2, alternately known as Requiem and Baf45D [48,49], is another mSWI/SNF subunit that, like Baf250A, is specific to the BAF complex [20]. The shRNA-mediated KD of *Dpf2* in C2C12 cells was achieved (Figure S2); these cells were compromised for myogenic differentiation as judged by myogenin and MHC staining and fusion index determinations (Figures S2 and S3), mirroring what was observed upon *Baf250A* KD.

**Figure 4:**
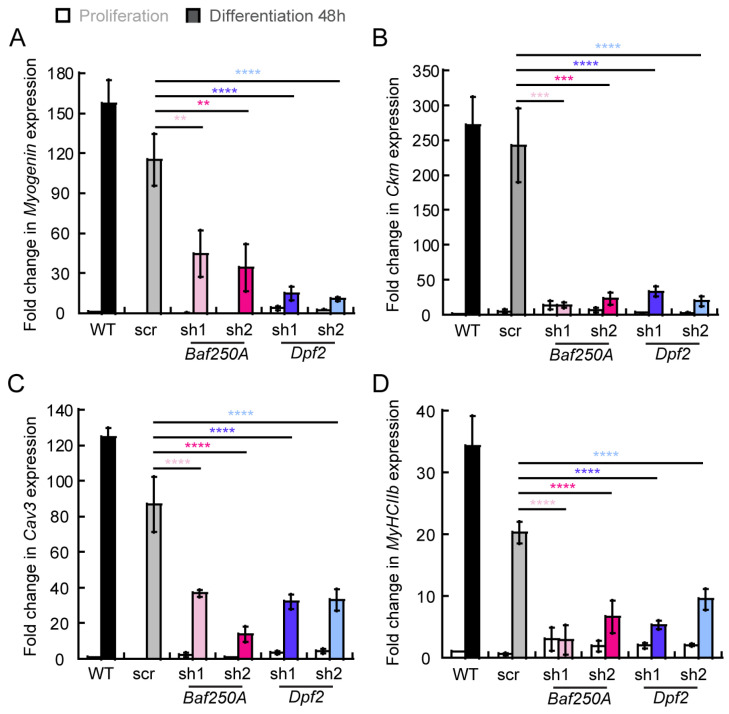
The expression of myogenic genes is impaired in differentiating myoblasts knocked down for the BAF complex subunits *Baf250A* and *Dpf2*. Steady-state mRNA levels of (**A**) *Myogenin*, (**B**) muscle-specific creatine kinase (*Ckm*), (**C**) Caveolin 3 (*Cav3*), and (**D**) myosin heavy chain II (*MhcIIb*) determined by qRT-PCR. Proliferating samples are represented by open bars. Samples differentiated for 48 h are represented by shaded bars. Among the differentiated samples, black represents wild type (WT) cells, gray represents scr shRNA treated cells, light and dark pink represent different shRNAs used for *Baf250A* KD, and light and dark blue represents different shRNAs used for *Dpf2* KD. mRNA levels were normalized against expression from the *Eef1A1* gene, which was used as a control. For each gene, the data were normalized against expression in proliferating control cells, which was set at 1.0, and represent the mean ± SE for three independent experiments. ** *p* < 0.01, *** *p* < 0.001, **** *p* < 0.0001.

We then validated expression of a few myogenic genes by quantitative reverse transcriptase PCR (qRT-PCR), in cells knocked down for *Baf250A* or *Dpf2*. We quantified steady-state mRNA levels of the differentiation markers myogenin (*Myog*), muscle specific creatine kinase (*Ckm*), caveolin 3 (*Cav3*), and myosin heavy chain IIb (*MyHCIIb*). We detected a significant decrease in the amount of each myogenic transcript in cells knocked down for *Baf250A* or *Dpf2* (Figure 4). 

**Figure 5:**
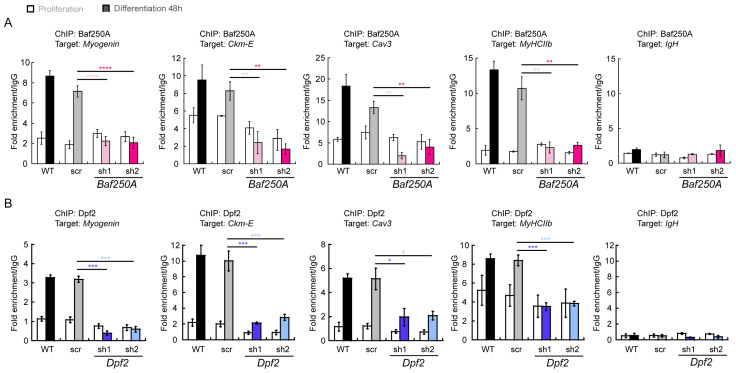
Effect of KD of BAF complex components on the binding of specific subunits to myogenic gene regulatory sequences. ChIP-qPCR showing binding of Baf250A (**A**) and Dpf2 (**B**) to the *Myogenin* promoter, the muscle creatine kinase enhancer (*Ckm-E*), the Caveolin 3 (*Cav3*) promoter, the myosin heavy chain II (*MhcIIb*) promoter, and the immunoglobulin H promoter (*IgH*; negative control) in proliferating and 48 h differentiating C2C12 myoblasts. Proliferating samples are represented by open bars. Samples differentiated for 48 h are represented by shaded bars. Among the differentiated samples, black represents wild type (WT) cells, gray represents scr shRNA treated cells, light and dark pink represent different shRNAs used for *Baf250A* KD, and light and dark blue represents different shRNAs used for *Dpf2* KD. Data are the mean ± SE for three independent experiments. * *p* < 0.05, ** *p* < 0.01, *** *p* < 0.001, **** *p* < 0.0001.

It is well established that the mSWI/SNF ATPases can be localized by ChIP methods to regulatory sequences controlling the expression of myogenic genes and that knockdown or inhibitors of the bromodomains located in the ATPase proteins can inhibit binding to target sequences [40]. Since the BAF complex is required for myoblast differentiation and activation of myogenic gene expression, we would predict that KD of BAF complex-specific subunits would deleteriously impact binding of the BAF enzyme to myogenic gene regulatory sequences. The data showed that there was a significant decrease in Baf250A binding to myogenic gene regulatory sequences in cells partially depleted of this subunit (Figure 5A). KD of the Dpf2 subunit similarly decreased binding to the myogenic gene regulatory sequences tested (Figure 5B). 

**Figure 6:**
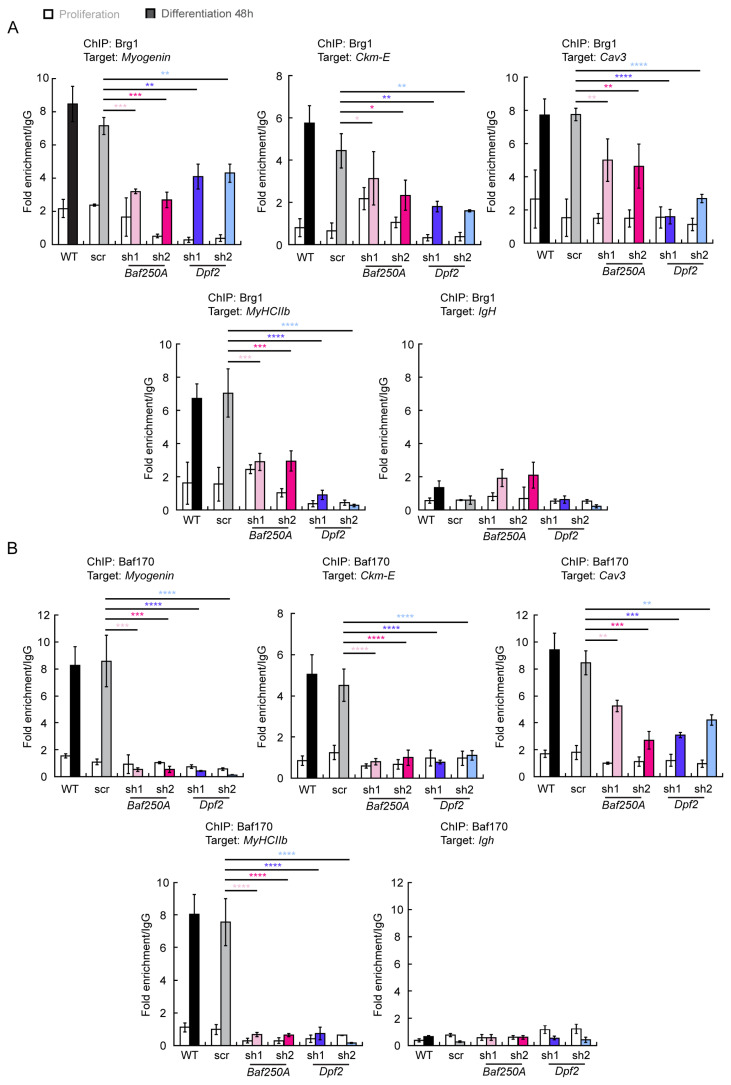
Binding of Brg1 and Baf170 to myogenic gene regulatory sequences is compromised upon KD of *Baf250A* or *Dpf2*. ChIP-qPCR showing binding to the indicated sequences of (**A**) Brg1 and (**B**) Baf170 in proliferating and differentiating C2C12 myoblasts. Proliferating samples are represented by open bars. Samples differentiated for 48 h are represented by shaded bars. Among the differentiated samples, black represents wild type (WT) cells, gray represents scr shRNA treated cells, light and dark pink represent different shRNAs used for *Baf250A* KD, and light and dark blue represents different shRNAs used for *Dpf2* KD. Binding in untransduced, differentiating C2C12 cells was shown for comparison. Binding to the *IgH* gene is shown as a negative control. Data are the mean ± SE for three independent experiments. * *p* < 0.05, ** *p* < 0.01, *** *p* < 0.001, **** *p* < 0.0001.

Prior work examining how mSWI/SNF complexes that belong to different sub-families assemble in solution has determined that the BAF-specific subunits, Baf250A and Dpf2, join after the “core” subunits are assembled into a sub-complex. The ATPase subunits join subsequently and are among the last of the subunits to join the complex [20]. We investigated whether KD of Baf250A or of Dpf2 proteins would impact the binding of the Brg1 ATPase that is critical for catalytic function. As expected, Brg1 binding was compromised if Baf250A or Dpf2 levels were reduced by KD (Figure 6A). In contrast, the Baf170 subunit is required to initiate the formation of the core complex that can exist in the absence of Baf250A or Dpf2 [20]. We investigated whether or not Baf170 was bound to myogenic regulatory sequences in the presence of *Baf250A* or *Dpf2* KD. We repeated the ChIP analysis for Baf170 and determined that its binding was also compromised at the target sequences (Figure 6B). KD of either BAF-specific subunit therefore compromises the ability of the BAF complex to interact with target sequences and does not support the idea that a partial BAF complex can stably bind.

## 3. Discussion

Cell and tissue lineage determination and differentiation are complex processes that rely on the tight regulation of gene expression by transcription factors, chromatin remodeling enzymes, and other co-regulatory proteins. Transcriptional regulators can be expressed in a tissue-specific manner to modulate the expression of tissue-specific genes to allow cells to differentiate into tissues and organs and to enable the growth and development of eukaryotes. Among these proteins, chromatin remodelers of the mSWI/SNF family drive developmental events that enable cells to proliferate and differentiate [50,51,52,53,54]. These enzymes utilize the energy released from ATP hydrolysis to modify the structure of nucleosomes and the chromatin environment at specific loci, depending on the cellular requirements [55,56,57]. The mSWI/SNF complex has been divided into three different families based on their subunit composition, though they are sometimes separated by the presence of the catalytic subunits, Brg1 or Brm [10,20,58]. 

In this work, we expanded our studies on the specific biological roles of the three sub-families of the mSWI/SNF complex by examining myoblast differentiation. Myogenesis is characterized by the expression of myogenic regulatory factors (MyoD, MRF4, MYF5 and myogenin) that bind to consensus E-boxes at the promoters and enhancers of myogenic genes [55,59,60,61]. These cooperate with the family of myocyte enhancer factor 2 (MEF2) proteins and other transcription factors to promote the expression of a downstream cascade of myogenic genes [62,63]. Recruitment of the mSWI/SNF enzymes by these transcription factors is required for activation of myogenic genes expressed both early and late in the differentiation process [26,27,28,30,31,64]. 

Here, we demonstrated that the BAF sub-family of mSWI/SNF enzymes, physically defined by the presence of the Baf250A and Dpf2 subunits, specifically mediates activation of the myogenic gene expression program. Knockdown of either protein inhibited myoblast differentiation, expression of representative myogenic marker genes, and binding of not just those subunits but other mSWI/SNF subunits at myogenic gene regulatory sequences. GO term analysis of genes that were downregulated in differentiating myoblasts subjected to *Baf250A* KD identified categories related to muscle development and structure, providing corroborating evidence that the BAF sub-family of mSWI/SNF enzymes contributes to myoblast differentiation. Our prior studies showed that the BAF sub-family was predominantly responsible for myoblast proliferation and for expression of the *Pax7* regulator of myogenic proliferation and viability [39]. This suggests an essential role for the BAF sub-family from at least the point of myoblast specification through myoblast formation. 

In contrast, KD of the Brd9 subunit, which is specific to the ncBAF complex, had a more modest effect on myoblast differentiation, and analysis of RNA-seq data did not identify terms related to muscle differentiation and function, suggesting that the impact of ncBAF complex disruption on myoblast differentiation is indirect. We speculate that the effects of Brd9 KD on myoblast differentiation are due to dysregulation of ribosomal biogenesis and rRNA processing, as seven of the top ten GO terms identified from the pool of genes upregulated in Brd9 KD cells related to rRNA, ribosomes, and translation. mSWI/SNF proteins recently have been linked to altered translational efficiency in cancer cells via direct mechanisms and via inhibition of mSWI/SNF protein function by chemical inhibitors [65,66]; it is possible that altering the gene expression and the processing of ribosome components also promotes altered translational efficiency.

The Baf180 protein, which is unique to the PBAF sub-family of mSWI/SNF enzymes, was not required for myoblast differentiation. The work here complements prior cell culture and in vivo studies indicating Baf180 is dispensable for myogenesis [40,41,42]. Prior work also demonstrated that Baf180, and by extension the PBAF complex, was dispensable for myoblast proliferation and for activation of the *Pax7* regulator of myoblast proliferation [39]. 

There is a paucity of information on the functional distinctions between the different sub-families of mSWI/SNF enzymes in development. We have presented evidence that the BAF sub-family is primarily responsible for maintaining myoblasts in the proliferative state via activation of the *Pax7* gene [39] and for initiating lineage-specific gene expression during differentiation. Similarly, there is a requirement for the BAF sub-family, but not the PBAF sub-family, in adipogenic differentiation [41]. In contrast, PBAF subunits are required for osteoblast differentiation [67,68], though the contribution of the ncBAF and BAF sub-families of enzymes is not clear. Baf250A is also required for cardiac precursor cell differentiation and for proper heart formation [69,70,71,72], but PBAF-specific subunits, including Baf180 and Baf200, are also required for heart formation [73,74,75]. This suggests that there are either separable requirements for the two sub-families or there is an uncharacterized mechanism of cooperation. Baf250A has also been implicated in neural stem cell proliferation and differentiation during cortical development [76], early embryo development, and ES cell differentiation [77,78,79]. However, the requirement for other mSWI/SNF sub-families was not determined for these processes. ncBAF was reported to regulate pluripotency in mouse ES cells [23], and the Brd9 component of ncBAF was required for pigment specific gene expression during melanocyte differentiation [80], but little else is known outside the context of cancer. At present, general conclusions about the roles of the different sub-families are not possible.

Studies addressing the mechanism of action of the mSWI/SNF chromatin remodelers suggest there may be stepwise assembly of the enzyme at target sequences. For instance, in proliferating myoblasts, the p38 kinase phosphorylates the BAF60c subunit of the chromatin remodeler and enables its association with MYOD and recruitment to myogenic gene promoters [30,64]. The phosphorylated BAF60c-MYOD complex acts as a scaffold that brings additional subunits of the mSWI/SNF complex to the myogenic loci to make chromatin accessible [30]. Later studies by Mashtalir et al. demonstrated that the components of the three different families are recruited sequentially and that the catalytic subunit is among the last components to associate in order to ultimately modulate gene expression programs [20]. Whether this programmed and organized association of the chromatin remodeler components is maintained on chromatin and/or in different tissues remains to be elucidated. However, results from this study and our prior studies suggest that knockdown of Baf250A or of Brg1, inhibition of Brg1 with the bromodomain inhibitor PFI-3, or inhibition of Brg1 with the calcineurin inhibitor FK506 inhibits the binding of other mSWI/SNF subunits [39,40,81,82], suggesting that stable subcomplexes of different configurations of mSWI/SNF enzymes with chromatin are not detectable by conventional ChIP methods. Additional work will be needed to distinguish between the possibility of stepwise assembly of mSWI/SNF enzymes on chromatin and binding of a pre-formed complex.

## 4. Materials and Methods

### 4.1. Antibodies 

Hybridoma supernatants were obtained from the Developmental Studies Hybridoma Bank (University of Iowa) against myogenin (F5D, deposited by W. E. Wright) and anti-myosin heavy chain (MHC; MF20, deposited by D. A. Fischman). Mouse anti-Brg1 (G-7; sc-17796) and normal rabbit IgG (sc-2027) were from Santa Cruz Biotechnologies (Dallas, TX, USA). The rabbit anti-Brd9 antibody was from Invitrogen (Waltham, MA, USA) (PA5-113488). The mouse anti-Brd9 antibody (1H8, CBMAB-0174-YC) was from Creative Biolabs (Shirley, NY, USA). The rabbit anti-PBRM (Baf180, A0334), -Baf250A (A16648), -vinculin (A2752), -Dpf2 (A13271), and -GAPDH (A19056) antibodies were from Abclonal Technologies (Woburn, MA, USA). Secondary antibodies used for Western blot were HRP-conjugated anti-mouse and anti-rabbit (31430 and 31460, respectively) and for immunofluorescence were the goat anti-rabbit IgG Alexa Fluor Plus 594 and the goat anti-mouse IgG Alexa Fluor Plus 488 (A32740 and A32723, respectively) that were obtained from Thermo Fisher Scientific (Waltham, MA, USA).

### 4.2. Cell Culture

C2C12 and HEK293T cells were purchased from ATCC (Manassas, VA, USA) and were maintained at sub-confluent densities in proliferation media containing Dulbecco’s modified Eagle’s medium (DMEM) supplemented with 10% fetal bovine serum (FBS) and 1% penicillin–streptomycin in a humidified incubator at 37 °C with 5% CO_2_. Differentiation of C2C12 cells was initiated after cells reached 80% confluence. Cell differentiation was induced with differentiation media (DMEM supplemented with 2% horse serum, 1% insulin–transferrin–selenium-A supplement (Invitrogen) and 1% penicillin–streptomycin) in a humidified incubator at 37 °C with 5% CO_2_. Samples of differentiated myoblasts were processed after 48 h of induction of differentiation, a time when early and late myogenic gene expression has been initiated and differentiation is in progress.

### 4.3. Virus Production for shRNA Transduction of C2C12 Cells 

Mission plasmids (Sigma, Kawasaki, Japan) encoding for two different shRNAs against specific subunits of the three sub-families of mSWI/SNF complexes BAF (Baf250A, Dpf2), ncBAF (Brd9), and PBAF (Baf180) (Table S3) were isolated by using the pure yield plasmid midiprep system (Promega, Madison, WI, USA) following the manufacturer’s instructions. The shRNA (15 µg) and the packing vectors pLP1 (15 µg), pLP2 (6 µg), and pSVGV (3 µg) were transfected using lipofectamine 2000 (Thermo Fisher Scientific) into HEK293T cells for lentiviral production. After 24 and 48 h, the supernatants containing viral particles were collected and filtered using a 0.22 µm syringe filter (Millipore, Burlington, MA, USA). Proliferating C2C12 myoblasts were transduced with lentivirus in the presence of 8 µg/mL polybrene and selected with 2 µg/mL puromycin (Invitrogen). 

### 4.4. RT-qPCR Gene Expression Analysis

RNA was purified from three independent biological replicates of proliferating and differentiated C2C12 myoblasts with TRIzol (Invitrogen) following the manufacturer’s instructions. cDNA synthesis was performed with 500 ng of RNA as template, random primers, and SuperScript III reverse transcriptase (Invitrogen) following the manufacturer’s protocol. Quantitative RT-PCR was performed with Fast SYBR green master mix on the ABI StepOne Plus Sequence Detection System (Applied Biosystems, Foster City, CA, USA) using the primers listed in Table S4. The delta threshold cycle value (ΔC_T_) was calculated for each gene and represents the difference between the CT value of the gene of interest and that of the *Eef1A1* reference gene. 

### 4.5. RNA-Sequencing Analysis 

Duplicate samples for RNA sequencing were purified from 48 h differentiating C2C12 myoblasts with TRIzol (Invitrogen) following the manufacturer’s instructions. Sample quality and concentration was determined at the Molecular Biology Core Lab at the University of Massachusetts Chan Medical School, Fragment Analyzer services. RNA library preparation and sequencing was performed by the BGI Americas Corporation (Cambridge, MA, USA). Briefly, libraries were sequenced using the BGISEQ-500 platform, and reads were filtered to remove adaptor-polluted, low quality, and high content of unknown base (N) reads. About 99% raw reads were filtered out as clean reads, which were then mapped to mouse reference genome mm10 using HISAT. Transcripts were reconstructed using StringTie [83], and novel transcripts were identified using Cufflinks [84]. All transcripts were then combined together and mapped to mm10 reference transcriptome using Bowtie2 [85]. Gene expression levels were calculated using RSEM [86]. DEseq2 [87] and PoissonDis [88] algorithms were used to detect differentially expressed genes (DEGs). GO analysis using DAVID (https://david.ncifcrf.gov/tools.jsp; accessed on 1 October 2022) was performed on DEGs to cluster genes into function-based and pathway-based categories.

### 4.6. Western Blot Analyses

C2C12 myoblasts were washed with PBS and solubilized with RIPA buffer (10 mM piperazine-N,N-bis(2-ethanesulfonic acid), pH 7.4, 150 mM NaCl, 2 mM ethylenediamine-tetraacetic acid (EDTA), 1% Triton X-100, 0.5% sodium deoxycholate, and 10% glycerol) containing protease inhibitor cocktail (Thermo Fisher Scientific). Protein content was determined by Bradford [89]. Samples (20 µg) were prepared for SDS-PAGE by boiling in Laemmli buffer. The resolved proteins were electro-transferred to PVDF membranes (Millipore). The proteins of interest were detected with the specific antibodies as indicated in the figure legends and above, followed by species-specific peroxidase conjugated secondary antibodies and chemiluminescent detection (Tanon, Abclonal Technologies).

### 4.7. Immunocytochemistry Analyses

C2C12 myoblasts (control and KDs) were fixed overnight in 10% formalin–PBS at 4 °C. Cells were washed with PBS and permeabilized for 10 min with PBS containing 0.2% Triton X-100. Immunocytochemistry was performed using the hybridoma supernatants from the Developmental Studies Hybridoma Bank (University of Iowa) against myogenin and MHC. Samples were developed with the universal ABC kit (Vector Labs, Newark, CA, USA) following the manufacturer’s protocol. 

### 4.8. Calculation of Fusion Index

The fusion index was calculated as the ratio of the nuclei number in C2C12 myocytes with two or more nuclei vs. the total number of nuclei as previously described [90]. Edges and regions that did not show good cell adhesion were discarded from the analysis. Three independent biological replicates were grown in 24-well plates, and cells were induced to differentiate as described above. Quantitative analysis was performed using ImageJ software v.1.8 ([91,92] National Institutes of Health, Bethesda, MD, USA).

### 4.9. Chromatin Immunoprecipitation Assays

Chromatin immunoprecipitation assays were performed as previously described [37,39,81,82,90]. Briefly, differentiating (48 h) C2C12 myoblasts were cross-linked with 1% formaldehyde (Ted Pella Inc., Redding, CA, USA) for 10 min at room temperature. Formaldehyde quenching was carried out with 125 mM glycine for 5 min. Crosslinked myoblasts were washed twice with ice-cold phosphate-buffered saline (PBS) supplemented with protease inhibitor cocktail and lysed with 1 mL of ice-cold buffer A (10 mM Tris HCl (pH 7.5), 10 mM NaCl, 0.5% NP-40, 0.5 µM dithiothreitol (DTT), and protease inhibitors) by incubation on ice for 10 min. The nuclei were pelleted by centrifugation at 3000× *g*, washed with 1 mL of buffer B (20 mM Tris HCl (pH 8.1), 15 mM NaCl, 60 mM KCl, 1 mM CaCl_2_, and 0.5 µM DTT). DNA was sheared by incubating the nuclei in 100 µL of buffer B supplemented with 1000 units of micrococcal nuclease (M0247S; NEB) for 30 min at 37 °C; the reaction was stopped by adding 5 µL of 0.5 M EDTA. Nuclei were pelleted and resuspended in 400 µL of ChIP buffer (100 mM Tris HCl (pH 8.1), 20 mM EDTA, 200 mM NaCl, 0.2% sodium deoxycholate, 2% Triton X-100, and protease inhibitors), sonicated for 10 min (medium intensity, 30 s on/30 s off) in a Bioruptor UCD-200 system (Diagenode, Denville, NJ, USA), and centrifuged at 21,000× *g* for 5 min. The length of the fragmented chromatin was between 200 and 500 bp as analyzed on agarose gels. ChIP was performed by incubating specific antibodies against Brg1, Brd9, Baf170, Dpf2, or Baf250A with each sample for 2 h at 4 °C. Anti-IgG ChIPs were included as negative controls. Immunocomplexes were recovered with 20 µL of magnetic Dynabeads (Thermo Fisher Scientific) after an overnight incubation at 4 °C. Three sequential washes with low-salt ChIP buffer followed with one final high-salt washing step were performed to eliminate unspecific binding. Complexes were eluted in 100 µL of elution buffer (0.1 M NaHCO_3_, 1% SDS) for 30 min at 65 °C, incubated with 1 µL of RNAse (0.5 mg/mL) for 30 min at 37 °C, and reverse cross-linked by addition of 6 µL of 5M NaCl and 1 µL of proteinase K (1 mg/mL) overnight at 65 °C. DNA was purified using a ChIP DNA Clean & Concentrator kit (Zymo Research, Irvine, CA, USA). Bound DNA fragments were analyzed by quantitative PCR using SYBR green master mix. Quantification was performed using the fold enrichment threshold cycle method 2^Δ(CT sample – CT IgG)^, and data are shown relative to the results determined for the IgG control. The primer sequences are listed in Table S4.

### 4.10. Statistical Analysis

Statistical analysis was performed using Kaleidagraph (Version 4.1) or Graph Pad Prism 7.0b. Multiple data point comparisons and statistical significance were determined using one-way analysis of variance (ANOVA). Experiments where *p* < 0.05 were considered statistically significant.

## Data Availability

The RNA-seq datasets are available at GEO. The accession number is: GSE196281.

## Supplementary Materials

The following supporting information can be downloaded at: https://www.mdpi.com/article/10.3390/ijms241411256/s1. Reference [93] is cited in the Supplementary Materials.
