# Supplementary material for: Differential Contributions of mSWI/SNF Chromatin Remodeler Sub-Families to Myoblast Differentiation
Source: Int J Mol Sci. 2023 Jul 9;24(14):11256. doi: 10.3390/ijms241411256 (PMC10378909; doi:10.3390/ijms241411256)
Supplement: Supplementary file 1 [file ijms-24-11256-s001.zip › Padilla-Benavides supplemental information IJMS - FINAL.pdf]

Differential contributions of mSWI/SNF chromatin remodeler sub-families to myoblast differentiation

Teresita Padilla-Benavides[1*#], Monserrat Olea-Flores[1,2#], Tapan Sharma[2], Sabriya A. Syed[2&], Hanna Witwicka[2%], Miriam D. Zuñiga-Eulogio[1,3], Kexin Zhang[1], Napoleon Navarro-Tito[3] and Anthony N. Imbalzano[2*]

[1]Department of Molecular Biology and Biochemistry, Wesleyan University, Middletown, CT, 06459, USA

[2]Department of Biochemistry and Molecular Biotechnology, University of Massachusetts Chan Medical School, Worcester, MA, 01605, USA

[3]Facultad de Ciencias Químico Biológicas, Universidad Autónoma de Guerrero, Guerrero, Mexico

*Correspondence: T.P.-B. tpadillabena@wesleyan.edu and A.N.I. Anthony.Imbalzano@umassmed.edu

[#] The authors contributed equally to this manuscript

[&] Present address: The Jackson Laboratory for Genomic Medicine, Farmington, CT 06032, USA

[%] Present address: Charles River Laboratories, Shrewsbury MA, 01545, USA

Emails and ORCID:

T.P.-B. tpadillabena@wesleyan.edu 0000-0002-4624-0822

M.O.-F. Monserrat.OleaFlores@umassmed.edu 0000-0003-1813-9062

S.A.S. sabriya.syed@jax.org 0000-0003-3776-3581

T.S. tapan.sharma@umassmed.edu 0000-0003-2495-2236

M.D.Z.-E. miriamzuniga@uagro.mx 0000-0003-2904-8350

N.N.-T. nnavarro@uagro.mx 0000-0003-4911-0545

H.W. hanna.witwicka@crl.com

K.Z. kzhang@wesleyan.edu 0000-0003-4504-0017

A.N.I. Anthony.Imbalzano@umassmed.edu_0000-0003-0744-3413

Figure S1

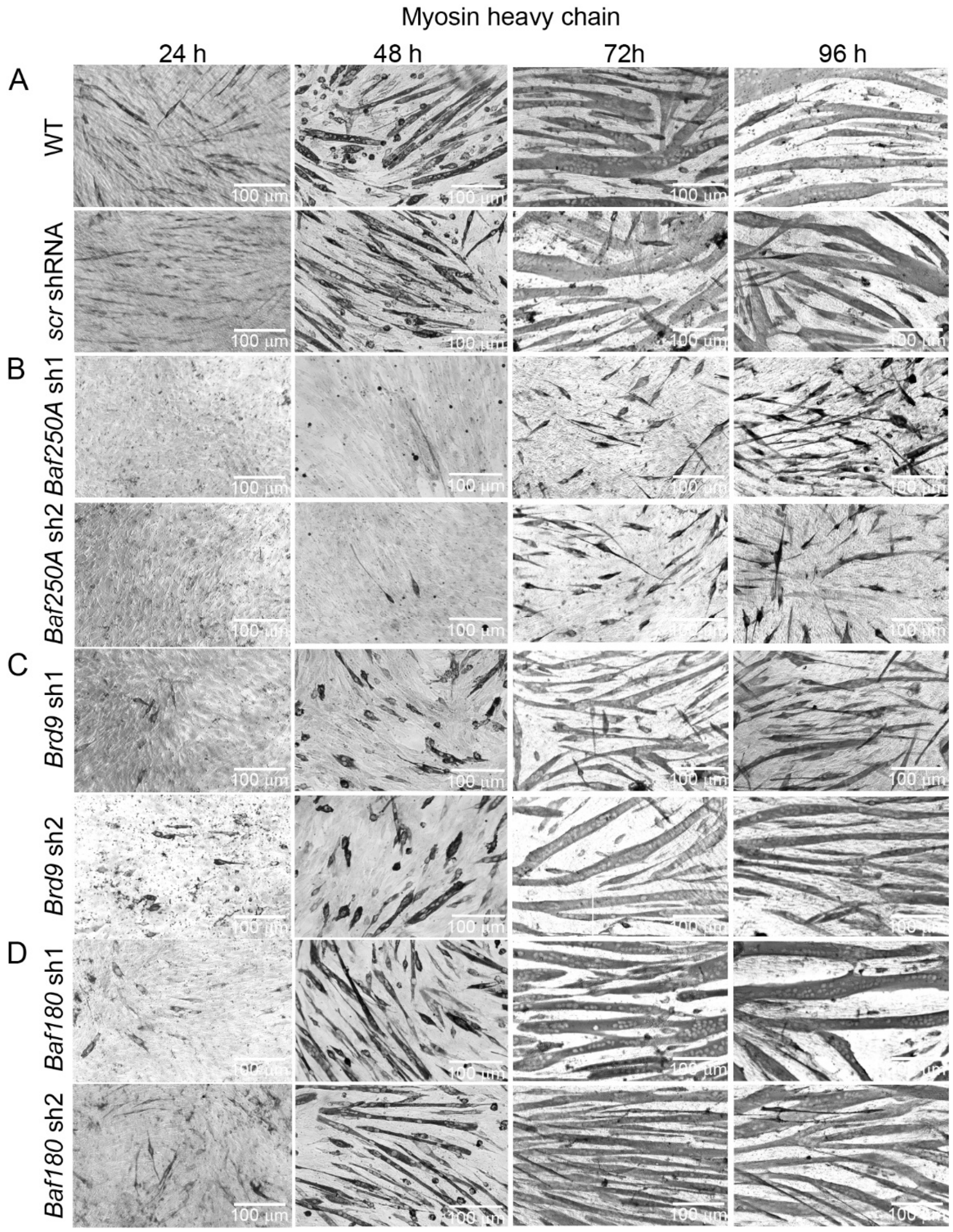

Figure S1. *Baf250A* knockdown inhibited the differentiation of C2C12 cells. Representative light micrographs of differentiating **(A)** wild type (WT) C2C12 myoblasts and cells transduced with *scr* shRNA **(B)** transduced with *Baf250A* shRNAs, **(C)** transduced with *Brd9* shRNAs, or **(D)** transduced with *Baf180* shRNAs. Cells at 24, 48, 72 and 96 h of differentiation were immunostained for myosin heavy chain. Bars = 100 µm.

Figure S2

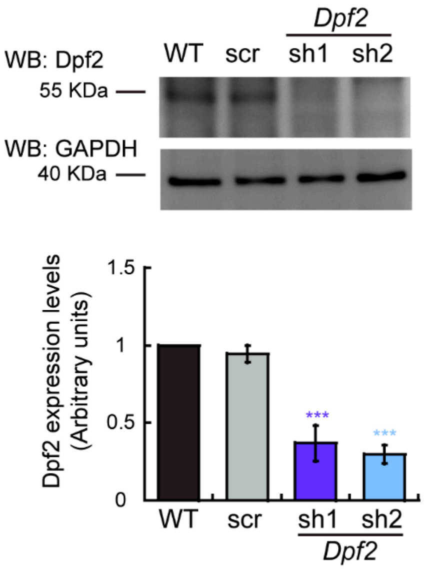

Figure S2. Expression levels of Dpf2 in differentiating wildtype (WT) C2C12 myoblasts. Representative immunoblots (top) and quantification of Dpf2 levels in myoblasts differentiating for 48 h. Immunoblots against GAPDH were used as loading controls. Samples were compared to the wild type sample, the value of which was set at 1.0. Data are the mean ± SE for three independent experiments. ***P < 0.001.

Figure S3

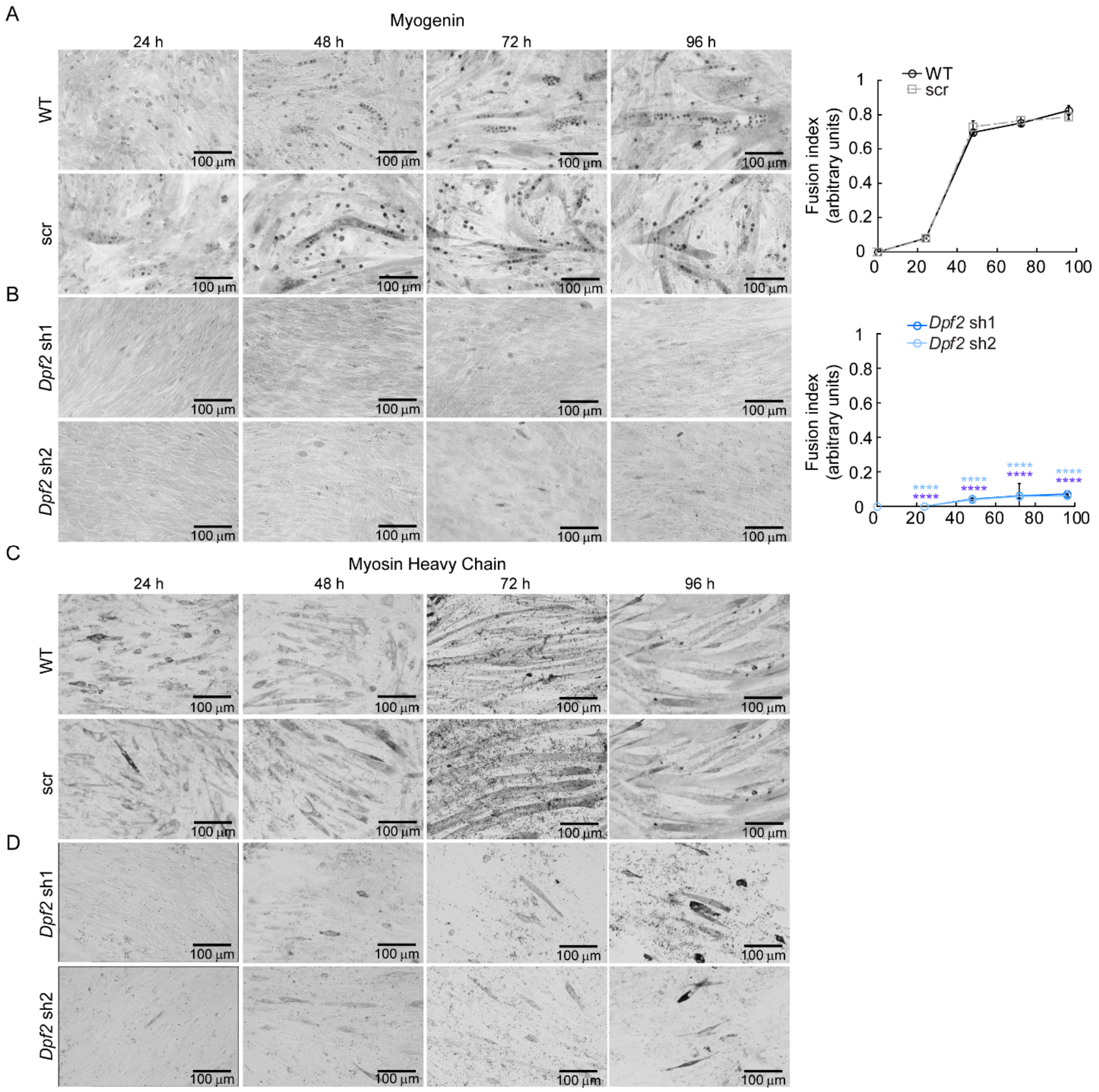

Figure S3. *Dpf2* knockdown inhibited the differentiation of C2C12 cells. Representative light micrographs and fusion index of differentiating **(A)** wild type (WT) C2C12 myoblasts and cells transduced with *scr* shRNA **(B)** transduced with *Dpf2* shRNAs. Cells at 24, 48, 72 and 96 h of differentiation were immunostained for myogenin and myosin heavy chain. Bars = 100 µm. **** P < 0.0001.

Table S1. Pearson coefficients for the two replicate samples for each KD RNA-Seq dataset

Sample	Pearson coefficient
scr shRNA differentiation	0.96
Baf250A shRNA differentiation	0.98
Brd9 shRNA differentiation	0.965
Baf180 shRNA differentiation	0.975

Table S3. Sequences of the shRNAs used in this study

Gene name	Sequence	Catalog number (Sigma)
Baf250 sh1	CCGGCTTTATAGTATGGCGAGTTAACTCGAGTTAACTCGCCATACTATAAAGTTTTTG	TRCN0000238304
Baf250 sh2	CCGGCCTAGGCAGCCTAACTATAATCTCGAGATTATAGTTAGGCTGCCTAGGTTTTTG	TRCN0000238306
Brd9 sh1	CCGGTGGACTTTGGCACGATGAAAGCTCGAGCTTTCATCGTGCCAAAGTCCATTTTTG	TRCN0000225737
Brd9 sh2	CCGGCACCGAATGGTGTCCAATAAGCTCGAGCTTATTGGACACCATTCGGTGTTTTTG	TRCN0000225739
Baf180 sh1	CCGGTGTGAAGTTGGTCCTAGTTTACTCGAGTAAACTAGGACCAACTTCACATTTTTG	TRCN0000304680
Baf180 sh2	CCGGGTGCAATATCCAGACTATTATCTCGAGATAATAGTCTGGATATTGCACTTTTTG	TRCN0000304681
Dpf2 sh1	CCTGGTGATTACAGGGTCAAA	TRCN0000084343
Dpf2 sh2	GCCTAACAACTACTGTGACTT	TRCN0000084345
scr shRNA pLKO.1-puro non-target shRNA control plasmid DNA	CCGGCAACAAGATGAAGAGCACCAACTCGAGTTGGTGCTCTTCATCTTGTTGTTTTT	MFCD07785395 SHC002

Table S4. List of primers used in this study

Primer name	5' sequence	3' sequence	Use	Reference
q-Myogenin	CAAGTGTGCACATCTGTTCTAGTCTCT	GTATCATCAGCACAGGAGACCTTGGT	Gene expression	Hernandez-Hernandez *et al*., 2013[1]
q-Ckm	CTGTCCGTGGAAGCTCTCAACAGC	TTTTGTTGTCGTTGTGCCAGATGCC	Gene expression	Hernandez-Hernandez *et al*., 2013[1]
q- MyHCIIb	TCAATGAGATGGAGATCCAGCTGAAC	GTCCAGGTGCAGCTGTGTGTCCTTC	Gene expression	Hernandez-Hernandez *et al*., 2013[1]
q-Cav3	TCAATGAGGACATTGTGAAGGTAGA	CAGTGTAGACAACAGGCGGT	Gene expression	Witwicka *et al*., 2019[2]
q-Eef1A1	GGCTTCACTGCTCAGGTGATTATC	ACACATGGGCTTGCCAGGGAC	Gene expression	Hernandez-Hernandez *et al*., 2013[1]
Myogenin promoter	ACGCCAACTGCTGGGTGCCA	GAATCACATGTAATCCACTGGA	ChIP qPCR	Hernandez-Hernandez *et al*., 2013[1]
Ckm enhancer	GACACCCGAGATGCCTGGTT	GATCCACCAGGGACAGGGTT	ChIP qPCR	Hernandez-Hernandez *et al*., 2013[1]
MyHCIIb promoter	CACCCAAGCCGGGAGAAACAGCC	GAGGAAGGACAGGACAGAGGCACC	ChIP qPCR	Hernandez-Hernandez *et al*., 2013[1]
Cav3 promoter	CCTAGGTGTCTCAGTCCAGTTA	CTGCCACGTAGATCTTGGAAAT	ChIP qPCR	Witwicka *et al*., 2019[2]
IgH enhancer	GCCGATCAGAACCAGAACACC	TGGTGGGGCTGGACAGAGTGTTTC	ChIP qPCR	Hernandez-Hernandez *et al*., 2013[1]

Supplemental references

1 Hernandez-Hernandez, J. M., Mallappa, C., Nasipak, B. T., Oesterreich, S. & Imbalzano, A. N. The Scaffold attachment factor b1 (Safb1) regulates myogenic differentiation by facilitating the transition of myogenic gene chromatin from a repressed to an activated state. *Nucleic Acids Res* **41**, 5704-5716, doi:10.1093/nar/gkt285 (2013).

2 Witwicka, H. *et al.* Calcineurin broadly regulates the initiation of skeletal muscle-specific gene expression by binding target promoters and facilitating the interaction of the SWI/SNF chromatin remodeling enzyme. *Mol Cell Biol*, doi:10.1128/MCB.00063-19 (2019).
